# PSA Distribution and Clinical Correlates in Azerbaijani Men Presenting to a Urology Clinic

**DOI:** 10.1155/proc/1854279

**Published:** 2026-09-07

**Authors:** Rashad Sholan, Rufat Aliyev, Ulduz Hashimova, Seymur Karimov, Elvin Bayramov

**Affiliations:** ^1^ Scientific Research Center, State Security Service Military Hospital, Baku, Azerbaijan; ^2^ Scientific Research Center, Azerbaijan Medical University, Baku, Azerbaijan, amu.edu.az; ^3^ A. Karayev’s Institute of Physiology, Minister of Science and Education, Baku, Azerbaijan; ^4^ Department of Kidney Diseases and Organ Transplantation, State Security Service Military Hospital, Baku, Azerbaijan; ^5^ Department of Laboratory, State Security Service Military Hospital, Baku, Azerbaijan

**Keywords:** Azerbaijan, prostate volume, prostate-specific antigen

## Abstract

**Background:**

Prostate‐specific antigen (PSA) levels are influenced by demographic and ethnic factors, but data from Azerbaijani men remain limited. PSA screening for prostate cancer must consider regional and ethnic variations. This study aimed to describe PSA levels and examine their clinical correlates in a hospital‐based cohort of Azerbaijani men presenting to a urology clinic.

**Methods:**

This retrospective cross‐sectional study included 1175 Azerbaijani men aged 40–75 years who presented to a urology clinic without a known diagnosis of prostate cancer between January 2023 and December 2024. Data collected included age, total PSA, digital rectal examination (DRE)–estimated prostate size category, prostate volume (via ultrasound), maximum urinary flow rate (*Q*_max_), and International Prostate Symptom Score (IPSS). Multivariate logistic regression was used to identify predictors of PSA elevation.

**Results:**

The median PSA level was 1.15 ng/mL (range: 0.001–112.1), with 85.1% of patients exhibiting levels below 4 ng/mL. PSA levels increased with age: 0.86 ng/mL in men aged 40–49, 1.2 ng/mL in those 50–64, and 1.44 ng/mL in men 65–75 years (*p* < 0.001). PSA correlated positively with age, IPSS, and prostate volume and negatively with *Q*_max_ (all *p* < 0.001). PSA levels varied with DRE‐estimated prostate size category, showing significant differences between Categories 2 and 3 (*p* = 0.002). Multivariate analysis identified age as an independent predictor of PSA ≥ 4 ng/mL.

**Conclusions:**

In this hospital‐based cohort of Azerbaijani men, PSA levels were positively associated with age, and age was independently associated with PSA ≥ 4 ng/mL. These findings provide important regional data and support further investigation of age‐specific PSA patterns in the Azerbaijani population.

## 1. Introduction

Prostate cancer remains a significant global health concern, representing the second most commonly diagnosed cancer in men worldwide [1]. While prostate cancer mortality rates have declined, likely due to a combination of factors including prostate‐specific antigen (PSA) screening and advancements in treatment, the incidence of metastatic disease has shown a concerning increase in recent years, particularly in older men [2–4]. This underscores the need for a better understanding of the demographic and clinical factors associated with PSA levels across different populations and clinical settings. While considerable research has investigated PSA screening in Western populations, data from other regions, including the Caucasus, are limited.

The complex relationship between PSA levels and prostate cancer is further complicated by the fact that PSA is not cancer‐specific. Benign prostatic hyperplasia (BPH), prostatic inflammation, and even perineal trauma can elevate PSA levels, potentially leading to false‐positive results and unnecessary interventions [5–7]. This highlights the challenge of accurately interpreting PSA results and the importance of considering patient‐specific factors.

This study aimed to describe the overall and age‐stratified distribution of total PSA; examine its associations with lower urinary tract symptoms, prostate size, and urinary flow parameters; and identify factors associated with PSA ≥ 4 ng/mL in a hospital‐based cohort of Azerbaijani men aged 40–75 years presenting to a urology clinic without a known diagnosis of prostate cancer.

## 2. Patients and Methods

This retrospective cross‐sectional study included 1175 Azerbaijani men aged 40–75 years who presented to the Urology Clinic of the State Security Service Military Hospital in Baku between January 2023 and December 2024. Patients with a known diagnosis of prostate cancer at the time of presentation and those with incomplete data for the variables of interest were excluded. Information on acute urinary retention, urinary tract infection, prostatitis, recent urological instrumentation, previous prostate surgery, ejaculation, and medications affecting PSA was not consistently available; therefore, these factors could not be applied as exclusion criteria. The study protocol was approved by the institutional ethics committee of the State Security Service Military Hospital, and the requirement for informed consent was waived due to the retrospective nature of the study.

Data collected included patient age, total PSA serum levels, prostate volume, digital rectal examination (DRE) findings, maximum urinary flow rate (*Q*_max_), and International Prostate Symptom Score (IPSS). DRE was used to obtain a subjective clinical estimate of prostate size. Based on the estimated volume on palpation, prostate size was categorized as follows: Category 1, ≤ 30 mL; Category 2, 31–60 mL; Category 3, 61–80 mL; and Category 4, > 80 mL. These categories reflected estimated prostate size only and did not represent a malignancy‐suspicion grading system based on nodularity, asymmetry, or induration. In contrast, ultrasonographic prostate volume was recorded as an objective continuous measurement and calculated from suprapubic ultrasonography (USG) using the prolate ellipsoid formula (length × width × height × *π*/6). *Q*_max_ was recorded as the peak urinary flow rate during uroflowmetry. Urinary symptoms were assessed using the validated seven‐question IPSS questionnaire, with scores ranging from 0 to 35, representing the severity of lower urinary tract symptoms. Based on established cutoff values, scores of 0–7 were classified as mild, 8–19 as moderate, and 20–35 as severe.

### 2.1. Statistical Analysis

Data were analyzed using SPSS Version 27 (IBM Corp., Armonk, NY, USA). Continuous variables were presented as mean ± standard deviation (SD) or median (range: min–max), and categorical variables as frequency (percentage). The 95% confidence intervals (CIs) for median values were calculated using nonparametric methods based on order statistics. Normality was assessed using visual methods and the Kolmogorov–Smirnov or Shapiro–Wilk tests. Categorical variables were compared using the chi‐square test. Continuous variables were analyzed with the Student′s *t*‐test or Mann–Whitney *U* test, based on distribution. Correlations between continuous variables were assessed using Spearman’s rank correlation. The Kruskal–Wallis test was used for comparing more than two nonparametric groups, with post hoc pairwise comparisons adjusted by Bonferroni correction. As a complementary threshold‐based analysis, factors independently associated with PSA ≥ 4 ng/mL were evaluated using multivariable logistic regression, including variables significant in bivariate analyses. A *p* value < 0.05 was considered statistically significant.

## 3. Results

**FIGURE 1:**
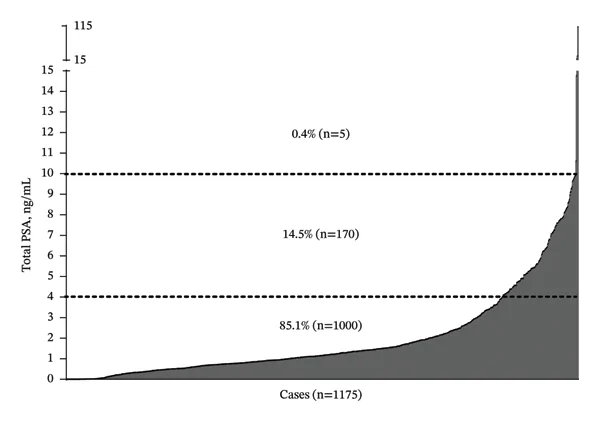
Distribution of total PSA levels among 1175 Azerbaijani men aged 40–75 years.

The study analyzed 1175 men with a mean age of 59.4 ± 9.5 years, 34.6% of whom were over 65 years. DRE‐estimated prostate size was ≤ 30 mL in 12.8% of participants, 31–60 mL in 69.0%, 61–80 mL in 6.0%, and > 80 mL in 12.3%. The median IPSS was 13 (range: 5–26), with 11.8% of patients experiencing mild, 72.9% moderate, and 15.2% severe lower urinary tract symptoms. The median total PSA level was 1.15 ng/mL (range, 0.001–112.1) with a 95% CI of 1.10 to 1.24 ng/mL, and 85.1% of patients exhibited levels below 4 ng/mL (Figure 1). Age‐stratified analysis showed median total PSA levels of 0.86 ng/mL (range, 0.02–7.8; 95% CI: 0.79–0.96) in men aged 40–49 years, 1.20 ng/mL (range, 0.001–112.1; 95% CI: 1.10–1.33) in those aged 50–64 years, and 1.44 ng/mL (range, 0.001–19.1; 95% CI: 1.24–1.79) in the 65–75‐year age group (*p* < 0.001). Ultrasound assessment showed a median prostate volume of 47 mL (range: 22–168), while uroflowmetry revealed a median Qmax of 13 mL/sec (range: 6–24).

**FIGURE 2:**
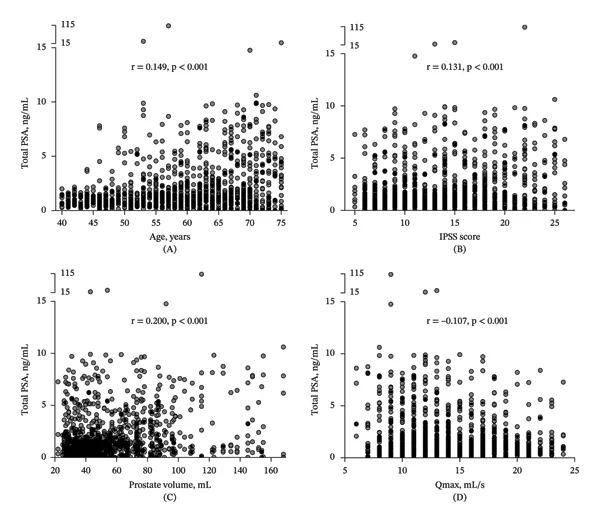
Correlations between total PSA levels with age (A), IPSS score (B), prostate volume (C), and *Q*_max_ (D).

**FIGURE 3:**
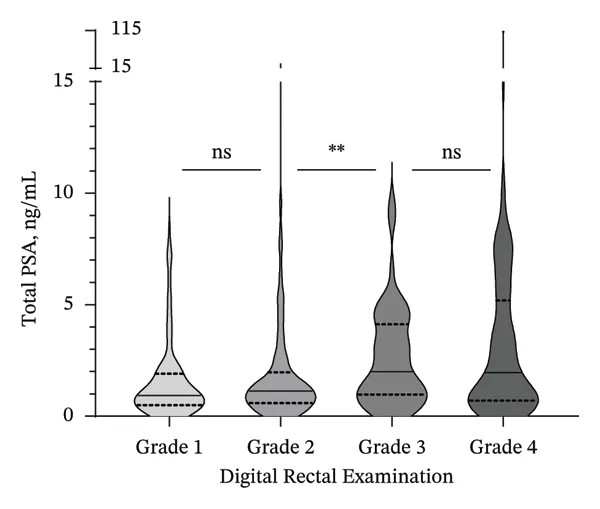
Distribution of total PSA levels by digital rectal examination categories (^∗∗^ *p* < 0.01).

Total PSA levels showed a positive correlation with age (*r* = 0.149, *p* < 0.001), IPSS score (*r* = 0.131, *p* < 0.001), and prostate volume (*r* = 0.200, *p* < 0.001) while demonstrating a negative correlation with Qmax (*r* = −0.107, *p* < 0.001) (Figure 2). The median total PSA level was 0.93 ng/mL (range: 0.001–8.4) in patients with DRE‐estimated prostate size Category 1, 1.13 ng/mL (range: 0.001–27.1) in Category 2, 2 ng/mL (range: 0.01–9.7) in Category 3, and 1.95 ng/mL (range: 0.001–112.1) in Category 4 (*p* < 0.001). Post hoc pairwise comparisons revealed no significant differences in total PSA levels between Categories 1 and 2 (*p* = 0.703) or between Categories 3 and 4 (*p* = 1.000). However, a significant difference was observed between Categories 2 and 3 (*p* = 0.002) (Figure 3).

Bivariate analyses revealed that total PSA levels ≥ 4 ng/mL were associated with older age, higher DRE‐estimated prostate size category, increased IPSS score, larger prostate volume, and lower Qmax (Table 1). In the multivariable logistic regression model, age was the only measured variable independently associated with PSA ≥ 4 ng/mL (Table 2).

**TABLE 1:** Study characteristics.

Characteristics	Total 1175 men	Total PSA		*p* value^∗^
		< 4 ng/mL, *n* = 1000	≥ 4 ng/mL, *n* = 175	
Age, mean ± SD, years	59.4 ± 9.5	58.5 ± 9.5	64.8 ± 7.4	**<** **0.001**

*Age categories, n (%)*				
40–49 years	226 (19.2)	221 (22.1)	5 (2.9)	**<** **0.001**
50–64 years	542 (46.1)	474 (47.4)	68 (38.9)	
65–75 years	407 (34.6)	305 (30.5)	102 (58.3)	

*DRE-estimated prostate size, n (%)*				
Up to ∼30 mL	150 (12.8)	135 (13.5)	15 (8.6)	**<** **0.001**
31 ∼ 60 mL	811 (69.0)	713 (71.3)	98 (56.0)	
61 ∼ 80 mL	70 (6.0)	51 (5.1)	19 (10.9)	
over ∼ 80 mL	144 (12.3)	101 (10.1)	43 (24.6)	
IPSS score, median (range)	13 (5–26)	13 (5–26)	16 (5–26)	**<** **0.001**

*IPSS symptom severity, n (%)*				
Mild (0–7 points)	139 (11.8)	127 (12.7)	12 (6.9)	**<** **0.001**
Moderate (8–19 points)	857 (72.9)	745 (74.5)	112 (64.0)	
Severe (20–35 points)	179 (15.2)	128 (12.8)	51 (29.1)	

Prostate volume, median (range) (mL)	47 (22–168)	46 (22–168)	65 (22–168)	**<** **0.001**

*Q*_max_ median (range) (mL/sec)	13 (6–24)	14 (6–24)	12 (6–24)	**<** **0.001**

*Note:* *Q*_max_, maximum urinary flow rate.

Abbreviations: DRE, digital rectal examination; IPSS, International Prostate Symptom Score; PSA, prostate‐specific antigen; SD, standard deviation.

^∗^Bold text indicates statistical significance at *p* < 0.05 level.

**TABLE 2:** Determination of independent predictors for PSA levels ≥ 4 ng/mL using multivariate logistic regression analysis.

Parameters	Odd’s ratio	95% CI	*p* value^∗^
Age	1.085	1.062–1.108	**<** **0.001**
DRE‐estimated prostate size > 60 mL (vs. ≤ 60 mL)	1.473	0.768–2.824	0.243
IPSS score	1.035	0.972–1.101	0.282
Prostate volume	1.010	0.999–1.021	0.080
*Q*_max_	1.031	0.959–1.108	0.415

*Note:* *Q*_max_, maximum urinary flow rate.

Abbreviations: CI, confidence interval; DRE, digital rectal examination; IPSS, International Prostate Symptom Score.

^∗^Bold text indicates statistical significance at *p* < 0.05 level.

## 4. Discussion

This study described total PSA levels and their associations with clinical parameters in 1175 Azerbaijani men aged 40–75 years who presented to a urology clinic without a known diagnosis of prostate cancer. Our findings provide descriptive data on PSA levels and their clinical correlates in a hospital‐based Azerbaijani cohort. They should not, however, be considered representative of healthy or asymptomatic men in the general Azerbaijani population.

Our key finding is that the median total PSA level in this population was 1.15 ng/mL, with 85.1% of men exhibiting levels below the conventional threshold of 4 ng/mL. This observation aligns with the growing recognition of significant interethnic variations in PSA levels [8, 9], highlighting the limitations of applying universal PSA cutoffs for prostate cancer screening and risk assessment. Prior studies in Iran [10] and Syria [9] have reported lower PSA values compared to Western populations, further supporting the need for population‐specific reference ranges. These geographic variations support further investigation of PSA distributions in representative Azerbaijani populations. [8, 10, 11]. However, direct comparisons between our findings and those from Western screening cohorts should be interpreted cautiously because of differences in age distributions, inclusion criteria, clinical settings, and methods of PSA assessment. In particular, our participants were men presenting to a urology clinic with urological complaints rather than healthy, asymptomatic individuals undergoing population‐based screening.

Our study demonstrated a positive correlation between total PSA levels, age, IPSS, and prostate volume, and a negative correlation with *Q*_max_. These findings are consistent with established knowledge regarding PSA physiology and the impact of age‐related prostatic changes [12, 13]. The age‐related increase in PSA is a well‐documented phenomenon, likely attributable to the cumulative effects of BPH and other age‐related prostatic changes. Furthermore, BPH and associated lower urinary tract symptoms, reflected by higher IPSS scores and decreased *Q*_max_, can also influence PSA levels.

While DRE remains a component of prostate evaluation, its diagnostic value, particularly in the screening setting, has been called into question. Our finding of statistically significant differences in PSA levels across different DRE‐estimated prostate size category suggests that DRE provides some clinical information relevant to prostate assessment. However, the lack of significant differences in post hoc comparisons between Categories 1 and 2 and Categories 3 and 4, coupled with the meta‐analysis by Matsukawa et al. [14], indicating limited independent values of DRE, highlights the need for caution when interpreting DRE findings and integrating them into clinical decision‐making.

In the multivariable analysis, age was the only measured variable independently associated with PSA ≥ 4 ng/mL. This finding is consistent with the age‐related increase in PSA reported in previous studies [11]. The U.S. Preventive Services Task Force recommends individualized decision‐making for men aged 55–69 regarding PSA screening, weighing potential benefits and harms, and discourages screening in men 70 and older [15]. Similarly, Nahvijou et al. [16] highlight the importance of tailoring screening based on prostate cancer risk and comorbidities. Current guidelines underscore the importance of educating patients about the potential benefits and harms of screening [17]. In this context, our study provides crucial data for informing these discussions in the Azerbaijani population.

This study has certain limitations. Its retrospective design introduces potential selection biases, and the single‐center setting may limit the generalizability of the findings to the broader Azerbaijani population. Several measurement‐related limitations should also be acknowledged. Information regarding the PSA assay platform, calibration standard, and exact timing of blood sampling relative to DRE or urological instrumentation was not available in the retrospective dataset. The absence of these technical details reduces the reproducibility of our findings and limits direct comparability with other established PSA cohorts. In addition, ultrasonographic and DRE assessments were performed during routine clinical care, and examiner‐level information and interobserver variability were not available. The DRE‐estimated prostate size categories were investigator‐defined and had not been formally validated. These factors may have introduced measurement variability and should be considered when interpreting the findings. Information on acute urinary retention, urinary tract infection, prostatitis, recent urological instrumentation, previous prostate surgery, ejaculation, and medications affecting PSA was not consistently available, resulting in potential residual confounding. Importantly, the participants were symptomatic men presenting to a urology clinic rather than randomly selected, asymptomatic members of the general population. The cohort may therefore have had a higher prevalence of benign prostatic enlargement, lower urinary tract symptoms, prostatitis, or bladder outlet obstruction, all of which can influence PSA levels. The observed PSA distribution should not be interpreted as a reference distribution for healthy Azerbaijani men. In addition, prostate biopsy and longitudinal follow‐up data were not systematically available. Therefore, some participants, particularly those with markedly elevated PSA levels, may have had undiagnosed prostate cancer. Prospective, multicenter studies incorporating biopsy results, PSA density, free‐to‐total PSA ratios, and longitudinal follow‐up are needed to clarify the clinical significance of PSA levels in Azerbaijani men. Furthermore, to avoid the loss of information associated with dichotomizing PSA at conventional thresholds, future research should model PSA as a continuous variable. Utilizing advanced statistical methods, such as log‐transformed multivariable linear regression or quantile regression, will better characterize the clinical determinants of PSA levels in this population. Population‐based studies that include asymptomatic participants are required before PSA reference ranges or screening thresholds can be proposed for the broader Azerbaijani population.

## Author Contributions

Conceptualization: Rashad Sholan; formal analysis: Rashad Sholan; funding acquisition: Rashad Sholan; investigation: Rashad Sholan, Ulduz Hashimova, Seymur Karimov, and Elvin Bayramov; methodology: Rashad Sholan, Rufat Aliyev, and Seymur Karimov; project administration: Rashad Sholan; resources: Rashad Sholan; supervision: Rufat Aliyev and Ulduz Hashimova; validation: Rufat Aliyev and Ulduz Hashimova; visualization: Rashad Sholan and Seymur Karimov; writing–original draft: Rashad Sholan and Seymur Karimov; writing–review and editing: Rufat Aliyev.

## Funding

The authors received no financial support for this article.

## Disclosure

All authors have read and agreed to the published version of the manuscript. An earlier version of this study was presented as a poster presentation at the 34th National Urology Congress, Türkiye.

## Ethics Statement

The study was approved by the State Security Service Scientific Research Center Ethics Board (Decision no: ETEK: 25/04).

## Conflicts of Interest

The authors declare no conflicts of interest.

## Data Availability

The data that support the findings of this study are available from the corresponding author upon reasonable request.

## References

[1] Bray F. , Laversanne M. , Sung H. et al., Global Cancer Statistics 2022: GLOBOCAN Estimates of Incidence and Mortality Worldwide for 36 Cancers in 185 Countries, CA: A Cancer Journal for Clinicians. (2024) 74, no. 3, 229–263, 10.3322/caac.21834. 38572751

[2] Etzioni R. , Tsodikov A. , Mariotto A. et al., Quantifying the Role of PSA Screening in the US Prostate Cancer Mortality Decline, Cancer Causes & Control. (2008) 19, no. 2, 175–181, 10.1007/s10552-007-9083-8. 18027095 PMC3064270

[3] Butler S. S. , Muralidhar V. , Zhao S. G. et al., Prostate Cancer Incidence Across Stage, NCCN Risk Groups, and Age Before and After USPSTF Grade D Recommendations Against Prostate-Specific Antigen Screening in 2012, Cancer. (2020) 126, no. 4, 717–724, 10.1002/cncr.32604. 31794057

[4] Desai M. M. , Cacciamani G. E. , Gill K. et al., Trends in Incidence of Metastatic Prostate Cancer in the US, JAMA Network Open. (2022) 5, no. 3, 10.1001/jamanetworkopen.2022.2246. PMC9907338 35285916

[5] Nadler R. B. , Humphrey P. A. , Smith D. S. , Catalona W. J. , and Ratliff T. L. , Effect of Inflammation and Benign Prostatic Hyperplasia on Elevated Serum Prostate Specific Antigen Levels, Journal d’Urologie. (1995) 154, no. 2 Pt 1, 407–413, 10.1016/s0022-5347(01)67064-2. 7541857

[6] Simardi L. H. , Tobias-MacHado M. , Kappaz G. T. , Taschner Goldenstein P. , Potts J. M. , and Wroclawski E. R. , Influence of Asymptomatic Histologic Prostatitis on Serum Prostate-Specific Antigen: A Prospective Study, Urology. (2004) 64, no. 6, 1098–1101, 10.1016/j.urology.2004.08.060. 15596176

[7] Oesterling J. E. , Rice D. C. , Glenski W. J. , and Bergstralh E. J. , Effect of Cystoscopy, Prostate Biopsy, and Transurethral Resection of Prostate on Serum Prostate-Specific Antigen Concentration, Urology. (1993) 42, no. 3, 276–282, 10.1016/0090-4295(93)90616-i. 7691013

[8] Mehrabi S. , Ghafarian Shirazi H. , Rasti M. , and Bayat B. , Analysis of Serum Prostate-Specific Antigen Levels in Men Aged 40 Years and Older in Yasuj, Iran, Urology Journal. (2005) 2, no. 4, 189–192. 17602427

[9] Bakir M. A. and Abo-daher D. , Age-Specific Reference Ranges for Prostate-Specific Antigen Among Healthy Syrian Men, International Journal of Biological Markers. (2012) 27, no. 2, e152–e159, 10.5301/jbm.2012.9304. 22653739

[10] Rahimifar S. and Montazeri A. , Age-Related Prostate Specific Antigen Reference Ranges in Healthy Northern Iranian Men, Iranian Journal of Immunology (IJI). (2018) 15, no. 1, 68–73, 10.22034/iji.2018.39342. 29549234

[11] Khezri A. A. , Shirazi M. , Ayatollahi S. M. et al., Age Specific Reference Levels of Serum Prostate-Specific Antigen, Prostate Volume and Prostate Specific Antigen Density in Healthy Iranian Men, Iranian Journal of Immunology (IJI). (2009) 6, no. 1, 40–48, 10.22034/iji.2009.17072. 19293477

[12] Ilic D. , Djulbegovic M. , Jung J. H. et al., Prostate Cancer Screening With Prostate-Specific Antigen (PSA) Test: A Systematic Review and Meta-Analysis, BMJ. (2018) 362, 10.1136/bmj.k3519. PMC6283370 30185521

[13] Dave P. , Carlsson S. V. , and Watts K. , Randomized Trials of PSA Screening, Urologic Oncology. (2025) 43, no. 1, 23–28, 10.1016/j.urolonc.2024.05.014. 38926075

[14] Matsukawa A. , Yanagisawa T. , Bekku K. et al., Comparing the Performance of Digital Rectal Examination and Prostate-Specific Antigen as a Screening Test for Prostate Cancer: A Systematic Review and Meta-Analysis, European Urology Oncology. (2024) 7, no. 4, 697–704, 10.1016/j.euo.2023.12.005. 38182488

[15] Grossman D. C. , Curry S. J. , Owens D. K. et al., Screening for Prostate Cancer: US Preventive Services Task Force Recommendation Statement, JAMA. (2018) 319, no. 18, 1901–1913, 10.1001/jama.2018.3710. 29801017

[16] Nahvijou A. , Hadian M. , and Mohamadkhani N. , Finding the PSA-Based Screening Stopping Age Using Prostate Cancer Risk, Cancer Treatment and Research Communications. (2024) 38, 10.1016/j.ctarc.2024.100791. 38266550

[17] Ali Asgari M. , Soleimani M. , Dadkhah F. et al., In the Era of Shared Decision Making, How Would an Iranian Urologist Screen Himself for Prostate Cancer?, Urology Journal. (2015) 12, no. 6, 2404–2409. 26706736

